# Impact of Immune Abnormalities on COVID-19 Vaccine Effectiveness in Infected Patients

**DOI:** 10.7759/cureus.74182

**Published:** 2024-11-21

**Authors:** Yunhui Chen, Wanxia Luo, Qiyu Huang, Yueming Chen, Weiping Yao

**Affiliations:** 1 Pulmonary and Critical Care Medicine, Foshan Sanshui District People's Hospital, Foshan, CHN; 2 Medical Services Section, Foshan Sanshui District People's Hospital, Foshan, CHN; 3 School of Public Health, Guangxi Medical University, Nanning, CHN; 4 Hospital Infection Management Section, Foshan Sanshui District People's Hospital, Foshan, CHN

**Keywords:** covid-19 vaccination, c-reactive protein (crp), d-dimer levels, immune statuses, ldh levels, sars-cov-2

## Abstract

Objective

To assess the protective effect of COVID-19 vaccines in patients with varying immune states by analyzing lactate dehydrogenase (LDH), C-reactive protein (CRP), and D-dimer (D-Di) levels in COVID-19-infected individuals under different vaccination scenarios and immune statuses.

Methods

This is a single-center retrospective study involving 338 SARS-CoV-2-infected patients treated at a tertiary medical center in Foshan, China, between November 2022 and January 2023. The primary outcome was the vaccine's protective effect on LDH, CRP, and D-Di levels.

Results

Vaccinated patients had shorter hospital stays and less severe lung involvement (P < 0.05), particularly in immunocompromised individuals. In immunocompromised patients, LDH and CRP levels were elevated, but in the vaccine group, fewer patients developed abnormally high CRP, D-Di, and coagulation-related complications. Vaccination reduced LDH and CRP levels in immunocompetent patients and lowered CRP levels regardless of immune status. In addition, people who are both immunocompromised and vaccinated have a higher risk of developing microthrombosis.

Conclusions

COVID-19 vaccination generally improves LDH, CRP, and D-Di levels, particularly in immunocompromised patients, supporting vaccination efforts. However, a subset of immunocompromised and vaccinated patients exhibited higher D-Di levels upon hospital admission, suggesting a complex interplay between vaccination, immune status, and the risk of microthrombosis. This finding emphasizes the need for further investigation into the role of immune function in disease severity and vaccine response.

## Introduction

As the COVID-19 pandemic persists as a global health crisis, the development, deployment, and rigorous evaluation of vaccine efficacy have taken center stage in public health strategies aimed at mitigating its spread [1]. A pivotal consideration in these efforts is the variability in vaccine protection observed across individuals, which is intimately tied to their immune status [2]. This underscores the urgent need to delve deeper into the widely discussed hypothesis that individuals with compromised immune function may experience diminished vaccine efficacy compared to those with normal immune responses [3-6].

We know that people living with HIV/AIDS are a particularly vulnerable group. Their compromised immune status may limit the body's ability to mount an effective antibody response following vaccination. This could result in reduced protection against SARS-CoV-2, the virus that causes COVID-19, compared to individuals with normal immune function. Studies have shown that HIV-positive patients may have lower antibody titers post vaccination and may be at increased risk of breakthrough infections, despite being fully vaccinated [7].

While vaccines have proven to be instrumental in shaping our response to previous pandemics, the nuances of their protective effects, especially in the context of COVID-19 and across diverse immune profiles, require meticulous examination. Specifically, we need to know whether the protective effect of vaccines in the real world on people with abnormal immune function is comparable to the protective effect on people with a sound immune system. This is crucial, as some argue that vaccinating immunocompromised individuals may yield limited benefits [3], whereas normal immune status individuals are more likely to experience optimal vaccine-induced protection. Therefore, a comprehensive analysis of how abnormalities in acquired immune function modulate the effectiveness of vaccine protection in COVID-19-infected patients is imperative to inform vaccination policies and practices.

Lactate dehydrogenase (LDH), a ubiquitous enzyme found in many tissues, is released into the bloodstream upon cellular damage [8]. In the context of viral infections, elevated LDH levels can serve as an indirect marker of tissue injury and metabolic stress, often correlating with disease severity. While LDH alone may not directly represent overall immune status, it provides valuable insights into the body's response to viral insult and the potential for organ damage [9]. C-reactive protein (CRP), on the other hand, is a highly sensitive marker of inflammation [10]. Its rapid increase in response to infection or tissue injury reflects the activation of the innate immune system [11]. Elevated CRP levels are thus indicative of an ongoing inflammatory process, which is a crucial aspect of the immune response to viral infections like COVID-19 [12]. D-dimer (D-Di), as a marker of coagulation system activation, plays a pivotal role in assessing the balance between coagulation and fibrinolysis [13]. Abnormalities in this balance can lead to hypercoagulability, a common complication in severe viral infections, including COVID-19. By monitoring D-Di levels, we can gain insights into the risk of thrombosis and other coagulation-related complications, which are particularly relevant in immunocompromised patients [14].

These indicators provide an objective picture of all aspects of the body's response to viral infection and the potential impact on clinical outcomes [15-17]. While lymphocyte levels consistently below 1*10^9/L may represent an abnormality in their acquired immune function [18], LDH, CRP, and D-Di offer complementary information about the systemic consequences of viral infections and the immune system's efforts to combat them. Considering this, our study aims to conduct a comprehensive analysis of the protective effect of COVID-19 vaccines in patients with varying immune statuses. Specifically, we will delve deeper into the differences in LDH, CRP, and D-Di levels among COVID-19 patients who are vaccinated and unvaccinated, as well as those with normal and compromised immune statuses at the time of hospital admission. By observing and comparing these clinical indicators across different vaccination scenarios and immune statuses, we aim to uncover distinct patterns that can provide a scientific basis for the application of vaccines in the prevention and control of SARS-CoV-2. Ultimately, our findings will contribute to the optimization of vaccination strategies, aiming to enhance the effectiveness of COVID-19 vaccination programs.

## Materials and methods

Participants and study design

This single-center retrospective study involved patients with SARS-CoV-2 infection who were treated and discharged on improvement between 7 November 2022 and 31 January 2023 at a major tertiary medical center in Foshan City, Guangdong, China. By screening all subjects who met the inclusion criteria between 7 November 2022 and 31 January 2023, participants were selected to ensure a representative sample of the SARS-CoV-2 patient population.

The inclusion criteria were as follows: (i) upon admission, patients tested positive for SARS-CoV-2 on the nucleic acid test; (ii) patients included in the study ranged in age from 18 to 100 years; (iii) participants had no previous medical history of respiratory system diseases; (iv) patients with clinical outcomes of improvement and discharge following hospitalization. The reason for this is that patients who experience a clinical outcome of death may have a higher number of comorbidities and exhibit extremely abnormal physiological and biochemical parameters, potentially influencing the results.

The exclusion criteria were established to ensure the purity of the study sample and to avoid potential biases as follows: (i) patients with moderate influenza (or high fever) or other pneumonia unrelated to SARS-CoV-2 infection were excluded, as these conditions could affect the interpretation of study outcomes; (ii) patients who received antiviral therapy with azvudine during hospitalization were not included, as this treatment could interfere with the assessment of natural disease progression and treatment response; (iii) patients without complete laboratory tests performed on admission were excluded, as these tests were essential for accurate patient characterization and outcome assessment.

**Figure 1:**
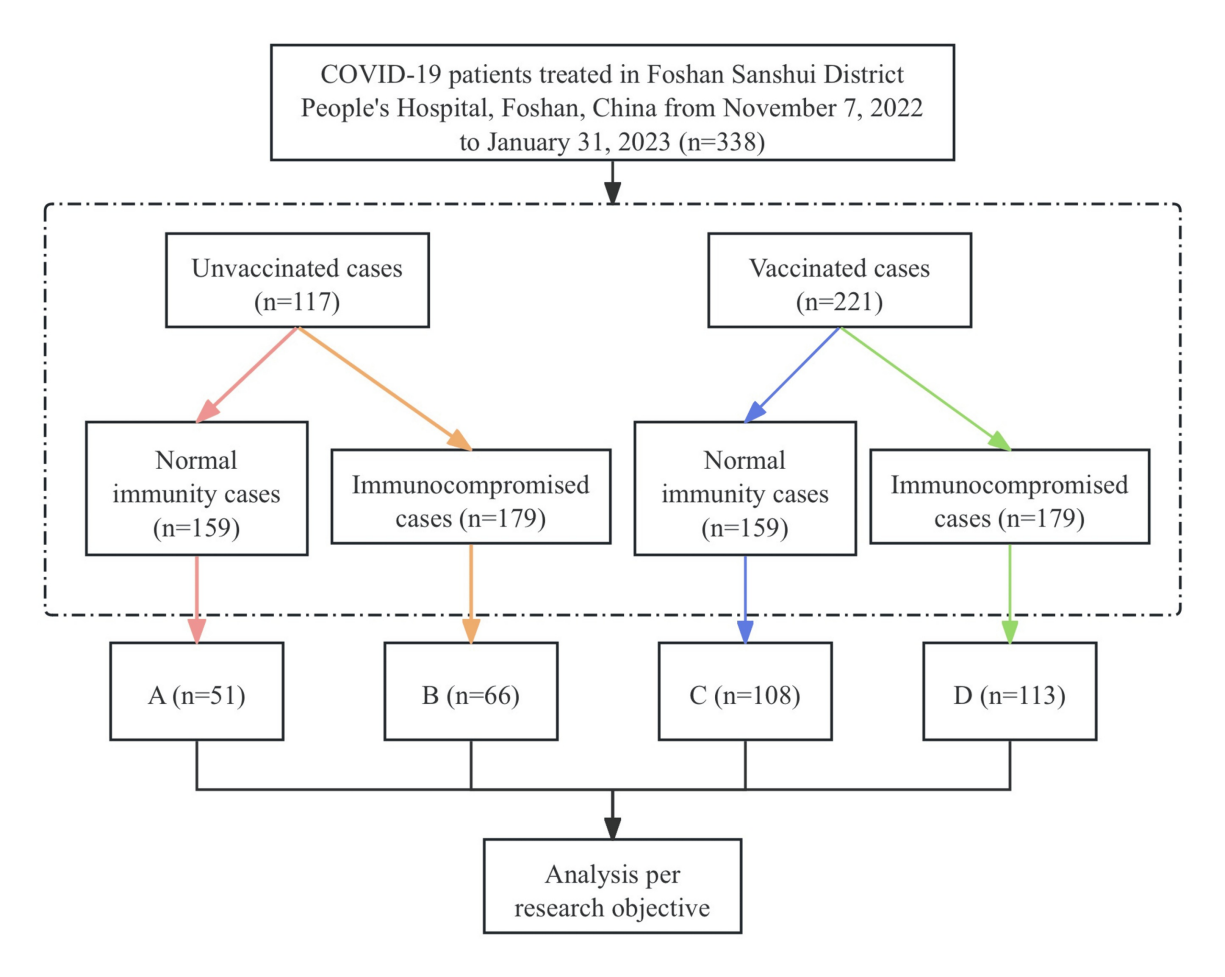
Patients and flow chart. COVID-19: coronavirus disease 2019; Normal immunity cases: patient's lymphocyte count on admission ≧ 1; Immunocompromised cases: patient's lymphocyte count on admission < 1. A: unvaccinated patients with normal immune function on admission; B: unvaccinated patients with abnormal acquired immune function on admission; C: vaccinated patients with normal immune function on admission; D: vaccinated patients but with abnormal acquired immune function on admission.

SARS-CoV-2 infection was defined as nasal swabs or saliva specimens that tested positive for both the ORF1ab gene and N gene by nucleic acid testing. The diagnosis of SARS-CoV-2 infection was in accordance with the guidelines of the World Health Organization (National Health Commission of the People's Republic of China, 2020). In this retrospective study, we classified patients infected with SARS-CoV-2 into four groups, i.e., A, B, C, and D, based on previous vaccination status and different immune statuses at admission (Figure 1). The different immune statuses of the patients were classified as immunocompetent (patient's lymphocyte count on admission ≧ 1 × 10^9/L) and immunocompromised (patient's lymphocyte count on admission <1 × 10^9/L) [18]. Our investigation focused on the impact of vaccine protection on the levels of LDH (with a normal range of 0-225 U/L), CRP (with a normal range of 0-10 mg/L), and D-Di (with a normal range of 0-550 mg/L) in COVID-19-infected patients with different immunological statuses. Notably, we analyzed LDH, CRP, and D-Di levels only at admission and did not conduct serial measurements over the hospitalization period. The defined normal ranges for these biomarkers apply to both adult males and females in the context of this study.

The study was reviewed and approved by the Ethics Committee of Foshan Sanshui People’s Hospital (Guangdong, China) (SRY-KY-2023046) and conformed to the ethical standards for medical research involving human subjects, as laid out in the 1964 Declaration of Helsinki and its later amendments. Participants provided written informed consent prior to taking part in this study. All the authors had access to the study's data and reviewed and approved the final manuscript.

Data collection

We queried our hospital's electronic medical records database and searched for patients with laboratory-confirmed SARS-CoV-2 virus infection from 7 November 2022 to 31 January 2023. We collected the patients' admission history and records of all tests performed at the time of admission. Then, we collected data on medical history and chronic diseases through medical records and history reviews.

All laboratory values on the day of admission and during hospitalization were collected from the electronic medical record database. Laboratory values assessed in the study included lymphocytes. Blood biochemicals included liver and renal function marker levels, CRP, LDH, and D-Di. Detailed information on chest CT results was also extracted. If the radiologist's report mentioned abnormal chest CT results, they were classified according to the proportion of typical COVID-19 infiltrating shadows in the lungs. Pulmonary CT was graded according to the area of typical COVID-19 pneumonia infiltration in the patient's lung images: (i) 0; (ii) <30%; (iii) 30%-50%; and (iv) >50%. All data were entered into a computerized database and cross-checked twice to ensure accuracy.

COVID-19 vaccination status was reported through a survey. Participants either provided identifying information for verification or directly uploaded an image of their vaccination card. The vaccine information collected included the dates of vaccination and the doses received. For the purpose of this study, individuals were considered vaccinated if they had received at least one dose of the vaccine. All vaccinated individuals had completed their vaccination regimen (which for the Chinese-made inactivated vaccines used in this study typically comprised of two doses, with some individuals potentially having received a booster dose) between 8 January 2021 and 16 January 2023. To guarantee the accuracy of vaccination data, particularly when reliant on patient-uploaded vaccination cards, all submitted images undergo a thorough review by trained personnel for authenticity and completeness.

Observation indicators

The primary observation of the study was the comparison of LDH, CRP, and D-Di levels between vaccinated and unvaccinated patients among SARS-CoV-2-infected patients with different immune statuses (normal immunity or immunocompromised) at the time of admission. The level of LDH usually correlates with the degree of cellular damage and is reflective of cellular damage due to viral infection. CRP is an acute time-phase reactive protein whose elevation is usually associated with an inflammatory response and can be used to assess the degree of inflammation in an infection. Then, elevated D-Di, a fibrin degradation product, may indicate abnormal coagulation or thrombosis and correlate with disease severity and prognosis.

The secondary objective of the study was to meticulously assess whether the individual's immune status exerted any influence on the protective effect of the vaccine and to determine how this influence manifested in specific clinical indicators. Furthermore, a thorough analysis was conducted to establish the correlations between the vaccination status (whether an individual was vaccinated or unvaccinated) and the levels of LDH, CRP, and D-Di at the time of admission. This analysis aimed to provide deeper insights into the vaccine's impact on clinical indicators among patients infected with the new coronavirus.

Statistical analysis

We compared LDH, CRP, and D-Di levels between vaccinated and unvaccinated patients infected with SARS-CoV-2 and exhibiting different immune statuses (normal immunity or immunocompromised) at the time of admission. To ensure the validity and reliability of the results, we employed parametric or non-parametric tests based on the distribution of the data. For normally distributed data, independent t-tests or ANOVA were used to assess significant differences in clinical indicators between vaccinated and unvaccinated groups, as well as across different immune statuses. In cases of non-normal distribution, non-parametric tests such as the Mann-Whitney U test or the Kruskal-Wallis test were applied. The means for age and length of stay were compared across groups using one-way ANOVA. Dichotomous variables or unordered multivariate variables for all subjects were analyzed using Pearson's chi-square test or the Wilcoxon rank-sum test. Then, multivariate regression analysis was performed to control for potential confounding factors and to further explore the independent effects of vaccination and immune status on the observed clinical indicators. This approach allowed us to isolate the specific impact of the vaccine, considering other variables that might influence the levels of LDH, CRP, and D-Di. Additionally, to further explore the nature of these interactions, we conducted subgroup analyses. Within the vaccinated and unvaccinated groups, we compared LDH, CRP, and D-Di levels between patients with normal immunity and those with immunocompromised status. This approach helped us to disentangle the specific effects of vaccination and immune status on the clinical indicators. Correlation analysis was also performed to investigate the relationship between vaccination status, immune status, and the levels of LDH, CRP, and D-Di. We used appropriate correlation coefficients (e.g., Spearman's or Pearson's) to quantify the strength and direction of these relationships. We considered results with P-values < 0.05 to be statistically significant. All analyses were performed using SPSS software version 26.0 (IBM Corp., Armonk, NY).

## Results

Baseline information and clinical presentation

Table 1 shows basic information for all participants, who had a mean age of 62.17 years (SD = 18.18) and a roughly balanced ratio of males and females. Among the 338 participants, 69.53% had underlying medical conditions. The average age of the vaccinated group was lower than that of the unvaccinated group at the same immunization status (P < 0.05).

**Table 1:** Demographic and baseline characteristics of patients in each group. F: ANOVA, χ²: chi-square test; P < 0.05 indicates statistical significance. ≧1: patient's lymphocyte count on admission ≧1 (10^9/L); <1: patient's lymphocyte count on admission <1 (10^9/L). Underlying disease: these include cardiovascular disease, hypertension, diabetes, inflammatory bowel disease, chronic kidney disease, chronic obstructive pulmonary disease, immunocompromised status, and cancer.

Variables	Total (n = 338)	Group				Statistic	P
		Unvaccinated		Vaccinated			
		≧1	<1	≧1	<1		
		A (n =51)	B (n = 66)	C (n = 108)	D (n = 113)		
Age (years), mean ± SD	67.12 ± 18.18	68.18 ± 21.68	71.94 ± 16.33	61.91 ± 18.36	68.82 ± 16.26	F = 5.072	0.002
Gender, n (%)						χ² = 5.179	0.159
Male	180 (53.25)	22 (43.14)	34 (51.52)	55 (50.93)	69 (61.06)		
Female	158 (46.75)	29 (56.86)	32 (48.48)	53 (49.07)	44 (38.94)		
Underlying disease, n (%)						χ² = 13.274	0.004
Yes	235 (69.53)	40 (78.43)	51 (77.27)	61 (56.48)	83 (73.45)		
No	103 (30.47)	11 (21.57)	15 (22.73)	47 (43.52)	30 (26.55)		

Then, we found that the median length of stay for patients in groups A, B, C, and D was about seven to 10 days (Table 2). The proportion of critically ill patients was relatively low, at about 25%. With normal immunocompetence on admission (group A versus group C), fewer patients in the vaccinated group than in the unvaccinated group had typical COVID-19 pneumonia-infiltrating lung shadows. At the same time, in the case of immunocompromised admission (group B versus group D), 10% more patients in the unvaccinated group than in the vaccinated group had lung shadows of more than 50%.

**Table 2:** Comparison of clinical data of SARS-CoV-2-infected patients in each group. F: ANOVA; χ²: chi-square test; -: Fisher exact; P < 0.05 indicates statistical significance. ≧1: patient's lymphocyte count on admission ≧1 (10^9/L); <1: patient's lymphocyte count on admission <1 (10^9/L).

Variables	Total (n = 338)	Group				Statistic	P
		Unvaccinated		Vaccinated			
		≧1	<1	≧1	<1		
		A (n = 51)	B (n = 66)	C (n = 108)	D (n = 113)		
Length of hospitalization (days), mean ± SD	8.62 ± 6.70	8.35 ± 5.16	7.86 ± 5.52	7.78 ± 5.60	9.97 ± 8.53	F = 2.443	0.064
Type of pneumonia, n (%)						χ² = 5.867	0.119
Non-critical	252 (74.6)	35 (68.6)	44 (66.7)	88 (81.5)	85 (75.2)		
Critical	86 (25.4)	16 (31.4)	22 (33.3)	20 (18.5)	28 (24.8)		
Secondary infection, n (%)						-	0.184
Yes	23 (6.8)	6 (11.76)	3 (4.55)	4 (3.70)	10 (8.85)		
No	315 (93.2)	45 (88.24)	63 (95.45)	104 (96.30)	103 (91.15)		
The proportion of infiltrating lung shadows in COVID-19, n (%)						-	0.011
0	64 (18.93)	6 (11.76)	12 (18.18)	27 (25.00)	19 (16.81)		
<30%	196 (57.99)	31 (60.78)	34 (51.52)	61 (56.48)	70 (61.95)		
30-50%	13 (3.85)	1 (1.96)	9 (13.64)	2 (1.85)	1 (0.88)		
>50%	35 (10.36)	10 (19.61)	5 (7.58)	9 (8.33)	11 (9.73)		
Other	30 (8.88)	3 (5.88)	6 (9.09)	9 (8.33)	12 (10.62)		

Comparison of LDH, CRP, and D-Di levels in different immunization statuses and vaccination scenarios

Firstly, as can be seen in Table 3, the proportion of immunocompromised patients with abnormally elevated LDH levels on admission was 12% greater than that of immunocompetent patients (P < 0.05). Then, 12% fewer people in the vaccine group had abnormally elevated CRP levels on admission than in the unvaccinated group. The number of immunocompromised patients with abnormally elevated CRP levels on admission was 11% greater than the number of immunocompetent patients. Notably, among immunocompromised patients, 16% fewer patients in the vaccine group had abnormally elevated D-Di levels on admission than in the unvaccinated group (P < 0.05). This could mean that the vaccine could lead to a lower risk of coagulation-related complications.

**Table 3:** Percentage of patients with elevated levels of LDH, CRP, and D-Di in different immune statuses and different vaccination scenarios. * Using the chi-square test, P < 0.05 indicates statistical significance. P1: significance of the unvaccinated group compared to the vaccinated group; P2: significance of the ≧1 group compared to the <1 group, P3: significance of comparison of groups A and C; P4: significance of comparison of groups B and D. ≧1: patient's lymphocyte count on admission ≧1 (10^9/L); <1: patient's lymphocyte count on admission <1 (10^9/L). LDH: lactate dehydrogenase; CRP: C-reactive protein; D-Di: D-dimer.

Laboratory test	Total (n = 338)	Unvaccinated (n = 117)	Vaccinated (n = 221)	≧1 (n = 159)	<1 (n = 179)	Group				P1	P2	P3	P4
						Unvaccinated		Vaccinated					
						≧1	<1	≧1	<1				
						A (n =51)	B (n = 66)	C (n = 108)	D (n = 113)				
LDH (U/L), n (%)										0.171	0.028	0.144	0.709
0-225	153 (45.30)	47 (40.2)	106 (48.0)	82 (51.6)	71 (39.7)	22 (43.10)	25 (37.90)	60 (55.60)	46 (40.70)				
>225	185 (54.70)	70 (59.8)	115 (52.0)	77 (48.4)	108 (60.3)	29 (56.90)	41 (62.10)	48 (44.40)	67 (59.30)				
CRP (mg/L), n (%)										0.025	0.048	0.262	0.055
0-10	128 (38.32)	35 (29.9)	93 (42.1)	69 (43.4)	59 (33.0)	19 (37.25)	16 (24.20)	50 (46.30)	43 (38.10)				
>10	210 (62.10)	82 (70.1)	128 (57.9)	90 (56.6)	120 (67.0)	32 (62.75)	50 (75.80)	58 (53.70)	70 (61.90)				
D-Di (mg/L), n (%)										0.003	0.002	0.091	0.012
0-550	107 (31.70)	23 (19.7)	84 (38.0)	66 (41.5)	41 (22.9)	15 (29.41)	8 (12.10)	51 (47.20)	33 (29.20)				
>550	231 (68.30)	94 (80.3)	137 (62.0)	93 (58.5)	138 (77.1)	36 (70.59)	58 (87.90)	57 (52.80)	80 (70.80)				

Secondly, Table 4 shows that among patients with abnormally elevated levels of LDH, CRP, or D-Di on admission, the mean levels of LDH and CRP on admission were lower in the vaccinated group than in the unvaccinated group among patients with the same immune status. In contrast, we found that among patients with abnormal acquired immune function on admission, D-Di levels were higher in the vaccinated group than in the unvaccinated group (P < 0.05).

**Table 4:** Comparison of values in patients with abnormally elevated levels of LDH, CRP, and D-Di at hospital admission. * Using the Kruskal-Wallis test, P < 0.05 indicates statistical significance. M: median; Q₁: 1st quartile; Q₃: 3rd quartile. P1: significance of overall comparisons; P2: significance of comparison of groups A and C; P3: significance of comparison of groups B and D. ≧1: patient's lymphocyte count on admission ≧1 (10^9/L); <1: patient's lymphocyte count on admission <1 (10^9/L). LDH: lactate dehydrogenase; CRP: C-reactive protein; D-Di: D-dimer.

Laboratory test	Total	Group				P1	P2	P3
		Unvaccinated		Vaccinated				
		≧1	<1	≧1	<1			
		A	B	C	D			
LDH, U/L, (n = 185), M (Q₁, Q₃)	287.00 (251.00, 356.00)	313.00 (263.00, 393.00)	297.00 (258.00, 353.00)	280.50 (253.75, 357.75)	267.00 (246.00, 329.50)	0.260	0.252	0.238
CRP, mg/L, (n = 210), M (Q₁, Q₃)	41.48 (20.57, 88.72)	43.14 (21.07, 72.95)	53.24 (17.49, 116.18)	33.95 (17.04, 50.63)	45.13 (26.93, 92.15)	0.045	0.152	0.825
D-Di, mg/L, (n = 231), M (Q₁, Q₃)	1800.00 (980.00, 3840.00)	1565.00 (1170.00, 2100.00)	1510.00 (927.50, 3070.00)	1520.00 (870.00, 2710.00)	2350.00 (1185.00, 6797.50)	0.009	0.822	0.018

Multifactor regression analysis

Although our univariate analysis indicated potential associations between various immune statuses and vaccines with disease severity, reduced inflammatory response, or decreased risk of microthrombosis, these findings must be interpreted with due caution. To further investigate these relationships, we conducted a detailed analysis examining the impact of different immune statuses and vaccination status on patients exhibiting abnormally elevated levels of LDH, CRP, and D-Di (Table 5). Notably, after adjusting for potential confounding variables, we observed that patients in the vaccinated group exhibited a reduced risk of abnormally elevated CRP levels, specifically, with a risk that was 0.610 times lower compared to the unvaccinated group. This suggests that vaccination may contribute to mitigating the risk of a severe inflammatory response. Additionally, we found that normal immune function (OR = 0.470, 95% CI: 0.270-0.810) and vaccination (OR = 0.490, 95% CI: 0.300-0.810) served as protective factors against abnormally elevated D-Di levels in patients infected with SARS-CoV-2. These findings align with previous studies, reinforcing the hypothesis that vaccination may play a crucial role in limiting the clinical progression of SARS-CoV-2 infection.

**Table 5:** Multifactorial analysis of the risk of elevated LDH, CRP, and D-Di levels on admission to the hospital. * Adjusted for age and underlying disease. Using one-way and multifactorial regression analyses, P < 0.05 indicates statistical significance. ≧1: patient's lymphocyte count on admission ≧1 (10^9/L); <1: patient's lymphocyte count on admission <1 (10^9/L). Interaction term: groups A, B, C, and D of the target group. LDH: lactate dehydrogenase; CRP: C-reactive protein; D-Di: D-dimer.

Variables	Crude OR (95%CI)	P^1^	Adjusted OR (95%CI)	P^2^
Elevated levels of LDH				
Vaccination (reference: unvaccinated)				
Vaccinated	0.860 (0.540 - 1.360)	0.520	0.920 (0.580 - 1.470)	0.734
immunity status (reference: <1)				
≧1	0.640 (0.410 - 0.990)	0.045	0.670 (0.43 - 1.040)	0.075
Interaction term (reference: A)				
B	1.244 (0.591 - 2.620)	0.565	1.237 (0.583 - 2.627)	0.580
C	0.607 (0.310 - 1.188)	0.145	0.697 (0.351 - 1.384)	0.302
D	1.105 (0.566 - 2.158)	0.770	1.135 (0.577 - 2.233)	0.713
Elevated levels of CRP				
Vaccination (reference: unvaccinated)				
Vaccinated	0.580 (0.360 - 0.940)	0.026	0.610 (0.370 - 0.990)	0.044
immunity status (reference: <1)				
≧1	0.650 (0.420 - 1.010)	0.057	0.690 (0.440 - 1.090)	0.109
Interaction term (reference: A)				
B	1.855 (0.834 - 4.127)	0.130	1.795 (0.804 - 4.008)	0.154
C	0.689 (0.348 - 1.362)	0.284	0.716 (0.358 - 1.433)	0.346
D	0.967 (0.488 - 1.914)	0.922	0.957 (0.482 - 1.900)	0.899
Elevated levels of D-Di				
Vaccination (reference: unvaccinated)				
Vaccinated	0.440 (0.260 - 0.760)	0.003	0.470 (0.270 - 0.810)	0.006
immunity status (reference: <1)				
≧1	0.460 (0.290 - 0.750)	0.002	0.490 (0.300 - 0.810)	0.005
Interaction term (reference: A)				
B	3.021 (1.164 - 7.839)	0.023	2.919 (1.117 - 7.626)	0.029
C	0.466 (0.229 - 0.948)	0.035	0.513 (0.249 - 1.060)	0.072
D	1.010 (0.489 - 2.088)	0.978	1.008 (0.484 - 2.098)	0.983

Synergistic effects of immune status and vaccination status on biochemical indicators

In our study, we investigated the synergistic effects of immune status (specifically lymphocyte subsets) and vaccination status on three key biochemical indices: LDH, CRP, and D-Di on admission. The results show that although there is no significant interaction between these two factors, the protective effect of the vaccine on these biochemical indicators is nevertheless influenced by the individual's immune status.

**Figure 2:**
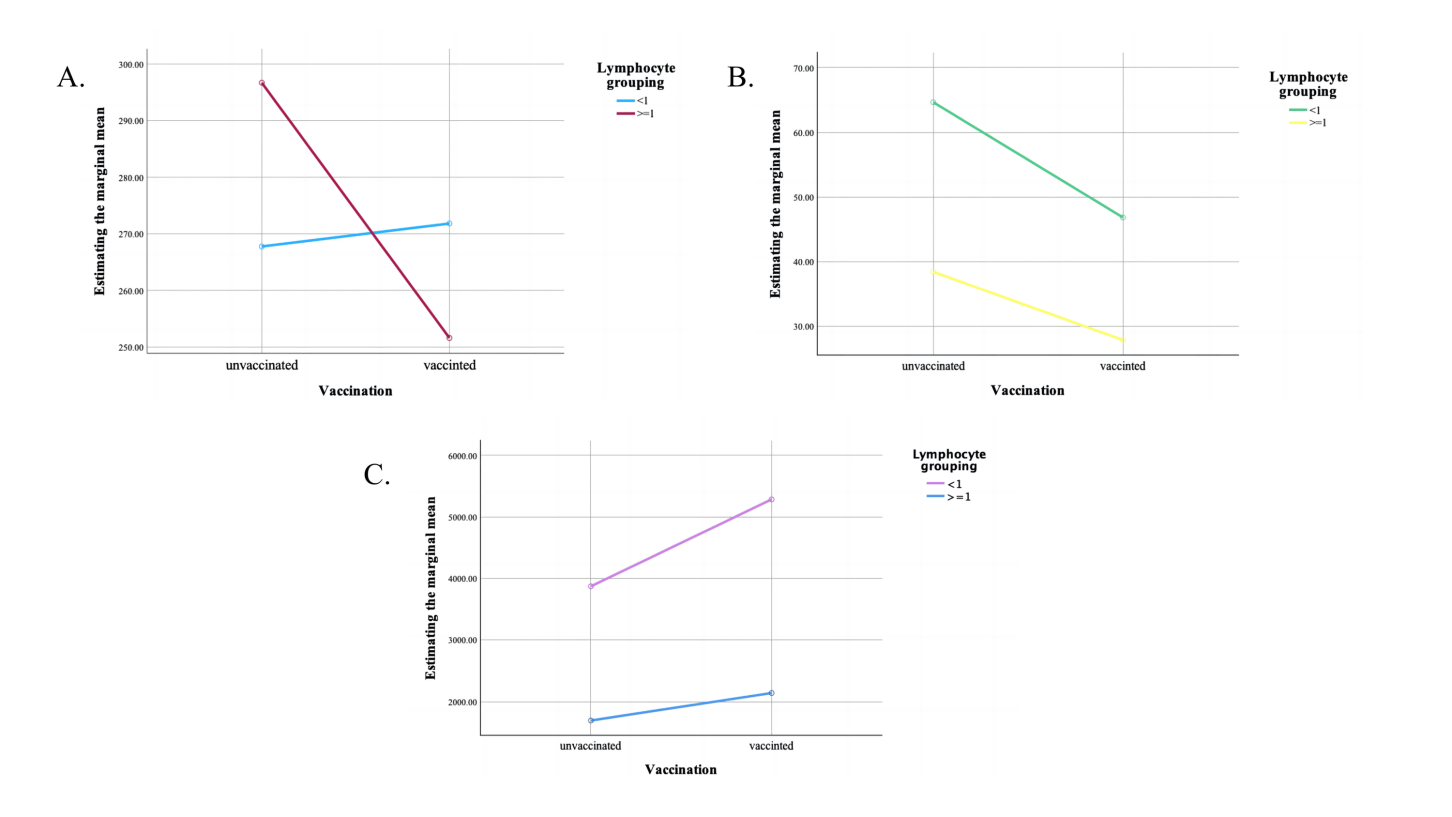
Interaction between vaccination and lymphocyte grouping (10^9/L) affecting LDH, CRP, and D-dimer levels on admission to the hospital. A: LDH (U/L); B: CRP (mg/L); C: D-dimer (mg/L). LDH: lactate dehydrogenase; CRP: C-reactive protein.

According to Figure 2A, we observed that in patients with a lymphocyte count greater than 1 (10^9/L) on admission (immunocompetent), the vaccine group had a more pronounced effect in reducing LDH levels on admission. Figure 2B shows that the inflammatory response was more intense in immunocompromised patients. Notably, the mean level of CRP was 10 units lower in the vaccine group than in the unvaccinated group, irrespective of an individual's immune status. We also found that although there was no interaction between vaccination status and individual immune status on admission, immunocompromised patients and patients in the vaccine group were more likely to develop microthrombi (Figure 2C).

These results suggest that the effect of vaccination on biochemical markers associated with COVID-19 disease severity is not homogeneous but is influenced by underlying immune status. Identifying this synergistic effect is critical to understanding the complex interplay between vaccination and the immune system in modulating disease outcomes.

## Discussion

Our study rigorously assessed the beneficial effects of vaccination on key clinical biomarkers (LDH, CRP, and D-Di) among COVID-19 patients, spanning a diverse range of immune statuses. The results highlight the crucial role of vaccination status in shaping clinical outcomes, particularly within the complex interplay of immune function.

Vaccination enhances immune responses in recipients, a well-established fact [19]. However, immune dysfunction in COVID-19 patients may compromise vaccine protection efficacy [20]. Achieving herd immunity, through vaccination or prior infection, remains crucial for controlling the spread of COVID-19. Studies on other vaccines, such as the pneumococcal vaccine, have shown effectiveness against community-acquired infections, especially in high-risk groups like HIV-infected patients [21]. Similarly, clinical biomarkers like IL-6, CRP, LDH, and D-Di have been identified as predictors of disease progression and treatment outcomes in COVID-19 [22]. Analysis of these biomarkers in vaccinated COVID-19 patients has also revealed a potential impact of vaccination [23].

Our investigation revealed a compelling trend among immunocompromised patients, characterized by lymphocyte counts below 1 × 10^9/L at admission. Specifically, unvaccinated SARS-CoV-2 patients with extensive lung involvement (defined as infiltrative shadows exceeding 30%) showed a significantly higher proportion compared to their vaccinated counterparts. This disparity underscores the potential of vaccination to mitigate severe lung manifestations in immunocompromised individuals, challenging the notion that such status precludes vaccination.

Our detailed examination of physiological and biochemical markers provided intriguing insights. Among patients with abnormal elevations of these markers upon admission, both immunocompetent and immunocompromised groups exhibited distinct patterns. In immunocompetent individuals, the unvaccinated cohort displayed significantly higher mean levels of LDH and CRP, with lower D-Di levels compared to the vaccinated group. Conversely, in immunocompromised patients, the vaccinated group showed elevated LDH and D-Di levels, albeit with lower CRP levels, than the unvaccinated group. This intricate interplay between immune status, vaccination status, and biomarker profiles underscores the complexity of the immune response to COVID-19 and emphasizes the pivotal role of vaccination in modulating this response.

Crucially, our initial observations indicated that, regardless of the patient's immune status at admission, the vaccinated group generally exhibited a lower prevalence of abnormal elevations in LDH, CRP, and D-Di compared to the unvaccinated group. However, upon further analysis, a nuanced picture emerged.

Specifically, for patients with compromised immune status at admission, we found that the vaccinated group had, unexpectedly, higher average levels of D-Di compared to their unvaccinated counterparts. This discrepancy was also observed in the median values for patients with abnormal D-Di elevations. These findings suggest a more complex interplay between vaccination status, immune status, and the inflammatory and coagulation cascades associated with COVID-19.

To further investigate this, we conducted a multifactorial regression analysis, which corroborated that vaccination emerged as a protective factor against the elevation of CRP and D-Di levels in COVID-19 patients in general. However, this protective effect seemed to be modulated by the patient's immune status, with immunocompromised patients experiencing a less pronounced benefit in terms of D-Di levels.

Additionally, our analysis consistently showed that irrespective of initial immune status, the vaccinated group had significantly lower proportions of individuals with abnormal elevations in LDH and CRP levels compared to the unvaccinated group (P < 0.05), suggesting a potential prophylactic effect of vaccination against certain aspects of the disease.

In delving deeper into the potential synergies between vaccination and immune status, we investigated their combined effects on LDH, CRP, and D-Di levels. Although no significant interaction was detected between these variables regarding LDH levels (P > 0.05), the protective benefit of vaccination was more pronounced among immunocompetent patients. Similarly, while no direct interaction was observed between vaccination and immunocompetence on CRP and D-Di levels, the inflammatory burden was generally mitigated in patients who were either vaccinated or immunocompetent. Conversely, immunocompromised patients exhibited more pronounced abnormalities in D-Di levels, emphasizing the pivotal role of immune function in modulating the coagulation cascade during COVID-19. These findings underscore the importance of vaccination, even in individuals with compromised immune systems, to mitigate the severe sequelae associated with the disease.

However, it is also crucial to acknowledge that our study possesses certain limitations. Firstly, the sample size may be inadequate to detect all conceivable associations, and the findings may not be universally applicable to all populations. Secondly, the study is observational in nature and is unable to establish definitive causal relationships between vaccination, immune status, and disease outcomes. Future studies will require larger sample sizes and more rigorous designs to confirm our observations and to explore in-depth the mechanisms underlying these associations.

## Conclusions

Our study provides evidence that COVID-19 vaccination is associated with improved clinical outcomes in terms of lung involvement and physiological biochemical marker levels, particularly in immunocompromised patients. These findings support the continued efforts to promote vaccination against COVID-19 and underscore the importance of immune function in modulating disease severity.
